# Real‐World Experience With Lenacapavir and/or Fostemsavir as Emerging Therapies for the Management of Multidrug‐Resistant HIV‐1 in Southeastern France

**DOI:** 10.1002/jmv.71149

**Published:** 2026-09-11

**Authors:** Syrine Boujamline, Celine Boschi, Hugo Bes‐Barlandier, Christelle Tomei, Amelie Menard, Saadia Mokhtari, Isabelle Ravaux, Yacine Belkhir, Nadege Neant, Caroline Solas, Philippe Colson, Matthieu Million

**Affiliations:** ^1^ IHU Mediterranée Infection Marseille France; ^2^ AP‐HM, Hôpital de la Timone Marseille France; ^3^ Aix‐Marseille Univ Microbes Evolution PHylogeny and Infection (MEPHI), UR D‐258 Marseille France; ^4^ Laboratoire de Pharmacocinetique et de Toxicologie Hôpital de la Timone, APHM Marseille France; ^5^ Unité des Virus Émergents (UVE: Aix‐Marseille Univ, Università di Corsica, IRD 190, Inserm 1207, IRBA) France

**Keywords:** antiretroviral therapy, antiviral drug resistance, fostemsavir, HIV‐1, lenacapavir, real‐world evidence

## Abstract

Lenacapavir and fostemsavir, two recently approved anti‐HIV‐1 agents, are promising options for people with difficult‐to‐treat HIV; however, real‐world data remain limited. This study aimed to describe the epidemiological, clinical, and virological profiles of people receiving combination antiretroviral regimens, including lenacapavir and/or fostemsavir. This retrospective study included adults living with HIV‐1 treated by lenacapavir and/or fostemsavir in our center (Marseille, France), between January 2023 and June 2024, with follow‐up continuing until July 2025. Clinical and biological data, including genotypic drug resistance testing and therapeutic drug monitoring, were extracted from electronic health records. The study included 21 people with HIV‐1 (14 men; mean age, 56 years), representing 1.0% of our active HIV cohort in 2023 (*n* = 2,126). Virological and immunological failure and treatment intensification were the main reasons for switching therapy. Before switching, viral load was detectable in 11 persons; 8 (73%) achieved undetectable viral load at 24 months, whereas 3 (27%) nonadherent people with lower CD4 nadirs, higher baseline viral loads, and lower baseline CD4 counts remained persistently viremic. All three developed high‐level genotypic resistance, including M426L and/or S375T for fostemsavir (two people) and Q67H, K70R, N74S, and/or A105AT for lenacapavir (two people). One developed resistance to both drugs. In one of these 3 persistently viremic PWH, a dual‐injectable rescue regimen combining lenacapavir and cabotegravir achieved viral suppression at 33 months with maintenance through the end of follow‐up, bringing the success rate to 9 out of 11 (82%) among PWH who were viremic at baseline. No severe lenacapavir‐ or fostemsavir‐related adverse events were observed. This small real‐world study supports the effectiveness and safety of lenacapavir and/or fostemsavir in people with multidrug‐resistant HIV and variable adherence. All adherent people achieved viral suppression, whereas drug‐resistant HIV‐1 emerged in three people with poor adherence. A dual‐injectable rescue regimen may represent a last‐resort option in selected complex cases. Similar real‐world studies should be expanded, and adherence support should be strengthened.

**Abbreviations**
AIDS: acquired immunodeficiency syndrome
ART: antiretroviral therapy
Ctrough: plasma trough concentration
FTR: fostemsavir
HIV: human immunodeficiency virus
HTE: heavily treatment‐experienced
INSTIs: integrase strand transfer inhibitors
LEN: lenacapavir
LLOQ: lower limit of quantification
NNRTIs: non‐nucleoside reverse transcriptase inhibitors
NRTIs: nucleoside reverse transcriptase inhibitors
PI: protease inhibitors
PWH: person living with HIV
TDM: therapeutic drug monitoring

## 1 Introduction

Despite major advances in antiretroviral therapy (ART), HIV infection remains a major public health concern worldwide, including in high‐income countries [1]. A subset of people with HIV (PWH) continue to experience virological failure (5%–10%) because of suboptimal adherence, cumulative drug resistance, long treatment histories, drug–drug interactions, intolerance, digestive malabsorption, or pharmacokinetic variability [2, 3, 4]. These challenges are particularly frequent in heavily treatment‐experienced (HTE) PWH, for whom the construction of a fully suppressive regimen may be difficult or impossible with conventional antiretroviral classes. In this context, antiretroviral agents with novel mechanisms of action are essential to restore viral suppression, preserve immune function, and prevent further resistance selection. In addition, combinations of at least two long‐acting injectable antiretrovirals, available since 2021, represent an attractive option for people with poor adherence to oral therapy or with malabsorption related to gut disorders, including AIDS‐associated enteropathy, particularly those with a history of low CD4 nadir [5, 6, 7].

Lenacapavir (LEN) is a first‐in‐class, long‐acting HIV‐1 capsid inhibitor that interferes with multiple stages of the viral life cycle, including capsid assembly, nuclear import, and virion production. Its pharmacological profile allows subcutaneous administration every 6 months after oral loading, making it particularly attractive for HTE PWH and for individuals in whom adherence to daily oral therapy is challenging. In the CAPELLA trial, viral suppression (HIV‐1 RNA < 50 copies/mL) was achieved in 81% of HTE PWH at week 26, 83% at week 52, and 62% at week 104 [8, 9]. In the CALIBRATE trial, lenacapavir combined with other antiretrovirals suppressed HIV‐1 viral load in up to 90% of treatment‐naïve PWH by week 54 [10]. However, trial populations are carefully selected, and the activity of LEN in everyday clinical practice may be influenced by incomplete adherence to companion drugs, delayed injections, residual activity of the optimized background regimen, and pre‐existing or emerging capsid mutations. Since 2025, only a few observational studies with limited sample sizes have been published, including two from the United States [11, 12], two from France [13, 14], and one from Italy [15]. Overall, these studies included only 203 PWH, underscoring the need for additional real‐world data.

Fostemsavir (FTR) is the prodrug of temsavir, an attachment inhibitor that binds directly to the HIV‐1 gp120 envelope glycoprotein, blocks interaction with the CD4 receptor, and thereby prevents viral entry. Because this mechanism differs from those of reverse transcriptase, protease, integrase, and fusion inhibitors, FTR may retain activity against viruses resistant to several conventional antiretroviral classes. In the phase 3 BRIGHTE trial, 65% of PWH in the randomized cohort achieved a ≥ 0.5 log_10_ reduction in viral load by day 8, compared with 19% in the placebo group [16]. By week 96, 60% had HIV‐1 RNA < 40 copies/mL, with a mean CD4^+^ T‐cell increase of 205 cells/mm^3^. The drug was generally well tolerated, with the most common adverse events being mild‐to‐moderate gastrointestinal symptoms, including nausea and diarrhea. Nevertheless, the clinical response to FTR may depend on the presence of gp120 substitutions affecting temsavir susceptibility, the number of active drugs in the background regimen, and sustained adherence to twice‐daily oral therapy.

Together, these findings indicate that LEN and FTR may be effective options for HTE PWH with limited antiretroviral options due to multidrug resistance, intolerance, drug–drug interactions, or contraindications to other classes. However, their optimal use in routine care remains incompletely defined, particularly in PWH with long‐standing infection, complex resistance patterns, advanced immunosuppression, and variable adherence. Real‐world studies are therefore needed to complement trial data by documenting treatment durability, virological and immunological outcomes, safety, therapeutic drug monitoring results, and the emergence of resistance under routine clinical conditions. We therefore aimed to evaluate the durability, effectiveness, safety, resistance emergence, pharmacological profile, and overall real‐world use of LEN and/or FTR in treatment‐experienced people living with HIV followed in Southeastern France.

## 2 Patients and Methods

### 2.1 Study Population, Study Period, and Ethical Considerations

After authorization by the Health Data Access Portal of Assistance Publique‐Hôpitaux de Marseille (AP‐HM; No. PADS25‐338) and approval by the AP‐HM Ethics and Scientific Committee (No. CSE25‐343), we conducted a retrospective cohort study at IHU Méditerranée Infection, AP‐HM, Marseille, Southeastern France. The study included adults aged ≥ 18 years living with HIV‐1 who had been prescribed lenacapavir, fostemsavir, or both between January 2023 and June 2024. Epidemiological, clinical, biological, virological, and therapeutic data were extracted from electronic health records stored in NADIS software and from the laboratory information system. Patients were informed that data recorded in NADIS could be used for research purposes and did not object. All data were fully anonymized. Follow‐up extended until death or the last recorded visit before the end of July 2025.

### 2.2 Virological Data

HIV‐1 RNA load was measured using either the NeuMoDx HIV‐1 Quant assay (Qiagen, Hilden, Germany; lower limit of detection, 32 copies/mL) or the GeneXpert HIV‐1 assay (Cepheid, Sunnyvale, CA, USA; lower limit of detection, 40 copies/mL). Detectable viral load was defined as plasma HIV RNA above 50 copies/mL. Hepatitis B virus (HBV) DNA and hepatitis C virus (HCV) RNA loads were measured using the NeuMoDx HBV and HCV Quant assays (Qiagen), respectively. HIV, HBV, and HCV serological testing was performed using Architect (Abbott Diagnostics, Lake Forest, IL, USA) or Cobas (Roche Diagnostics, Indianapolis, IN, USA) assays.

To characterize resistance mechanisms to lenacapavir and fostemsavir, we performed partial *gag* and *env* gene sequencing, respectively. Plasma was extracted from 200 µL of whole blood collected in EDTA tubes using the EZ1 Virus Mini Kit v2.0 on an EZ2 instrument (Qiagen, Hilden, Germany). The *gag* and *env* genes were amplified separately using in‐house protocols and specifically designed primers. First‐round PCR amplification was performed using SuperScript II One‐Step RT‐PCR with Platinum Taq polymerase (Invitrogen, Carlsbad, CA, USA), followed by nested PCR amplification using FastStart Taq DNA polymerase (Roche Applied Science, Indianapolis, IN, USA). For the HIV‐1 capsid region, a 1485‐bp fragment was amplified using the first‐round forward primer F2NSt_F1b (CGGAGGCTAGAAGGAGAGAGATG; positions 770–792 in the HIV‐1 HXB2 reference genome, GenBank accession No. K03455) and reverse primer 1448L_R1b (GGTCGCTGCCAAAGAGTGAT; positions 2260–2278), with annealing at 61°C for 15 s and extension at 68°C for 2 min. Second‐round PCR used forward primer *gag*_5U_F2 (GTGCGAGAGCGTCAATATTAAGAG; positions 794–815) and reverse primer 1445L_R2 (GGTCGCTGCCAAAGAGTGATT; positions 2260–2278), with annealing at 61°C for 15 s and extension at 72°C for 2 min. For the HIV‐1 *env* gene, a 1337‐bp fragment was amplified using first‐round forward primer *env*_B_R1_Fb (GAAAGAGCAGAAGACAGTGGCAAT; positions 6203–6226) and reverse primer *env*_M_R1_Rb (CCTTCCAGTCCCCCCTTTTC; positions 9073–9092), followed by second‐round forward primer F2*Env*_JR_R2_F (GTCTATTATGGGGTACCTGTGTGG; positions 6336–6359) and reverse primer CD4R_R2_R (AATTCACTTCTCCAATTGTCCCTC; positions 7649–7672). PCR amplicons were sequenced by next‐generation sequencing using Illumina technology and the Nextera XT kit with 2 × 250‐bp paired‐end reads on a MiSeq instrument (Illumina Inc., San Diego, CA, USA). *gag* and *env* sequences were reconstructed using an in‐house Python script [17], which assembles NGS reads by mapping them to the HIV‐1 HXB2 reference genome. Detection threshold used here to consider mutations within the quasispecies at a given nucleotide position of the HIV‐1 genome was 5% of NGS reads. Resistance mutations associated with lenacapavir and fostemsavir were interpreted using the French ANRS‐MIE algorithm (https://hivfrenchresistance.org/), the Stanford HIV Drug Resistance Database (https://hivdb.stanford.edu/) [18], and the HIV‐GRADE application, respectively [19]. Multidrug‐resistant HIV was defined as resistance to at least two antiretroviral drugs from at least three of the four main classes: nucleoside reverse transcriptase inhibitors, non‐nucleoside reverse transcriptase inhibitors, protease inhibitors, and integrase strand transfer inhibitors. HIV‐1 subtype was determined by phylogenetic analysis of the obtained HIV‐1 *gag* and *env* sequences, as described in the Supporting Material. The genotypic susceptibility score (GSS) was calculated according to the French ANRS‐MIE algorithm, assigning scores of 1, 0.5, and 0 for susceptible, intermediate‐resistant, and resistant antiretroviral drugs, respectively.

### 2.3 Treatment Adherence and Plasma Drug Trough Concentrations

Adherence to antiretroviral therapy was assessed by physicians using clinical judgment based on patient interviews and review of medical history. Plasma trough concentrations (C_trough_) of lenacapavir, temsavir, and associated antiretroviral drugs were collected when available as part of therapeutic drug monitoring (TDM). Drug concentrations were measured using a validated liquid chromatography–tandem mass spectrometry method [20] updated to include recently approved antiretrovirals, in accordance with the same validation criteria, with intra‐ and inter‐day precision and accuracy below 15% for all drugs. The lower limits of quantification were 5 ng/mL for lenacapavir (precision, 5.6%; accuracy, 0.76%) and 50 ng/mL for temsavir (precision, 7.5%; accuracy, 0.33%). Concentrations were interpreted using published therapeutic ranges [21, 22] and the recommended lenacapavir threshold of 15.5 ng/mL, corresponding to the four‐fold protein‐adjusted IC_95_ for wild‐type HIV‐1.

### 2.4 Statistical Analysis

Continuous variables were expressed as mean ± standard deviation and analyzed using the nonparametric Kruskal–Wallis test. Categorical variables were compared using the Chi‐square test or Fisher's exact test, as appropriate according to data distribution and expected cell counts. Three groups were defined: patients with virological suppression at baseline (Group A), viremic patients who achieved viral suppression by 24 months (Group B), and viremic patients who did not achieve viral suppression by 24 months (Group C). A *p* value < 0.05 was considered statistically significant. Statistical analyses were performed using XLSTAT, the Microsoft Excel data analysis add‐on (https://www.xlstat.com/en/), and GraphPad Prism (GraphPad Software, Boston, MA, USA; www.graphpad.com).

## 3 Results

### 3.1 Baseline Characteristics

Twenty‐one people with HIV (PWH) were included, representing 0.99% of our active cohort of 2126 PWH seen at least once in 2023. Detailed baseline characteristics at treatment switch are shown in Table 1. Three participants were born outside France: one in Spain, one in Algeria, and one in Cape Verde. The cohort included 14 men and seven women (male‐to‐female ratio, 2:1), all living with HIV‐1. Mean age was 54 years (range, 27–77 years), and 15 PWH (71%) were aged 50 years or older; the most represented age group was 60–69 years (48%). Mean duration of HIV infection since diagnosis was 31 years (range, 8–39 years). Eight PWH (38%) had received zidovudine monotherapy as first‐line treatment between 1992 and 1995 and therefore had experienced the full history of antiretroviral therapy. HIV transmission occurred through injecting drug use in seven PWH (33%), heterosexual contact in six (29%), mother‐to‐child transmission in four (19%), and homosexual contact and transfusion in two each (10%). Most PWH (67%) had at least one comorbidity. Dyslipidemia was the most common comorbidity, affecting nine PWH (43%), whereas hypertension and chronic cardiac disease each affected eight PWH (38%). Nine PWH were HCV‐seropositive, all with undetectable plasma HCV RNA, indicating past infection. Two PWH had serological markers of both past HCV and HBV infections, with undetectable plasma HCV RNA, negative hepatitis B surface antigen, and positive anti‐hepatitis B core antibody. Mean age at HIV diagnosis was 24 years (range, 0–40 years), including four PWH infected through mother‐to‐child transmission.

**TABLE 1:** Baseline characteristics.

Characteristics	All PWH (*n* = 21)	Group A virologically suppressed at baseline (*n* = 10)	Group B viremic at baseline with viral suppression by 24 months (*n* = 8)	Group C viremic at baseline without viral suppression by 24 months (*n* = 3)	*p* value
Age	53.9 ± 14.8	58.1 ± 15.2	54.5 ± 13.2	38.2 ± 8.7	0.117
Male sex (no. (%))	14 (67%)	7 (70%)	5 (63%)	2 (67%)	0.945
Country of birth					
France	18 (86%)	8 (80%)	8 (100%)	2 (67%)	0.200
Other country	3 (14%)	2 (20%)	0 (0%)	1 (33%)	
Comorbidities					
Hypertension	8 (38%)	5 (50%)	3 (38%)	0 (0%)	0.294
Diabetes	6 (29%)	4 (40%)	2 (25%)	0 (0%)	0.389
Dyslipidemia	9 (43%)	7 (70%)	2 (25%)	0 (0%)	0.043
Chronic renal disease	2 (10%)	2 (20%)	0	0	0.296
Chronic cardiac disease	8 (38%)	3 (30%)	4 (50%)	1 (33%)	0.675
Stroke	0 (0%)	0	0	0	1.000
Cancer	3 (14%)	0	2 (25%)	1 (33%)	0.192
Liver disease	2 (10%)	1 (10%)	1 (13%)	0	0.818
HIV disease characteristics					
Route of infection					0.185
Intravenous drug use	7 (33%)	2 (20%)	5 (63%)	0	
Heterosexual contact	6 (29%)	4 (40%)	0	2 (67%)	
Mother‐to‐child transmission	4 (19%)	1 (10%)	2 (25%)	1 (33%)	
Homosexual contact	2 (10%)	2 (20%)	0	0	
Transfusion	2 (10%)	1 (10%)	1 (13%)	0	
Duration of infection (months)	31.2 ± 8.6	30.1 ± 9.1	35.2 ± 3.9	24.0 ± 12.5	0.116
CDC stage					0.611
A	6 (29%)	2 (20%)	3 (38%)	1 (33%)	
B	7 (33%)	4 (40%)	3 (38%)	0	
C	8 (38%)	4 (40%)	2 (25%)	2 (67%)	
Viral hepatitis					
HCV history^a^	9 (43%)	4 (40%)	1 (13%)	0 (0%)	0.145
HBV (anti‐HBc+)	2 (10%)	1 (10%)	1 (13%)	0 (0%)	1.000
HIV subtype					0.571
B	20 (57%)	10 (100%)	8 (100%)	2 (66%)	
C	1 (5%)	0	0	1 (33%)	
Immunovirological parameters					
Nadir CD4 (cells/µL)	101.9 ± 115.0	121.1 ± 144.8	113.6 ± 78.6	6.3 ± 6.1	0.027
At baseline					
Viral load (copies/mL)	13 682 ± 32 295	27 ± 9	14 790 ± 23 416	56 242 ± 69 687	< 0.001
Persons with > 1000 copies/mL (no. (%))	8 (38%)	0 (0%)	5 (63%)	3 (100%)	0.001
Persons with > 100 000 copies/mL (no. (%))	1 (5%)	0 (0%)	0 (0%)	1 (33%)	0.071
CD4 (cells/µL)	465 ± 419	645 ± 450	397 ± 341	50 ± 42	0.013
CD4 category (cells/µL)					0.138^a^
< 200	8 (38%)	2 (20%)	3 (38%)	3 (100%)	
200–500	6 (29%)	3 (30%)	3 (38%)	0 (0%)	
> 500	7 (33%)	5 (50%)	2 (25%)	0 (0%)	
Treatment					
Number of pills before switch	4.0 ± 2.9	4.8 ± 3.4	3.0 ± 2.2	4.0 ± 2.6	0.491
Number of injectables before switch	0.2 ± 0.5	0.1 ± 0.3	0.2 ± 0.7	0.7 ± 0.6	0.117
Number of pills after switch	3.0 ± 2.2	2.2 ± 1.2	4.2 ± 2.9	2.3 ± 1.5	0.232
Number of injectables after switch	1.0 ± 0.7	1.0 ± 0.5	0.9 ± 0.8	1.3 ± 1.2	0.632
Reason for switch					
Virological failure	6 (29%)	0	4 (50%)	2 (67%)	0.269
Therapeutic intensification	3 (14%)	3 (30%)	0	0	
Immunological and virological failure	3 (14%)	1 (10%)	1 (13%)	1 (33%)	
Simplification	2 (10%)	2 (20%)	0	0	
Clinical side effects	2 (10%)	2 (20%)	0	0	
Pharmacological adaptation	2 (10%)	1 (10%)	1 (13%)	0	
Other (person decision, intolerance, etc.)	3 (14%)	2 (20%)	2 (25%)	0	

^a^ None had detectable HCV viral load. Group C is small (*n* = 3), which limits the statistical power of some comparisons.

CDC stage was A in six PWH (29%), B in seven (33%), and C in eight (38%). The mean CD4 nadir was 102 cells/mm^3^ (range, 1–489), and 17 PWH had a CD4 nadir < 200 cells/mm^3^. The mean most recent CD4 cell count was 465 cells/mm^3^ (range, 11–1375); eight PWH (38%) had CD4 counts < 200 cells/mm^3^, whereas seven (33%) had CD4 counts > 500 cells/mm^3^. HIV‐1 subtype, determined from the reverse transcriptase gene, was subtype B in 20 PWH (95%) and subtype C in one. Phylogenetic trees based on gag and env sequences are provided in the Supporting Material.

Before switching to a new antiretroviral regimen containing lenacapavir and/or fostemsavir, the mean HIV RNA load was 13 682 copies/mL (4.1 log_10_ copies/mL). Eight PWH had HIV RNA loads > 1000 copies/mL (3.0 log_10_), and one had a viral load > 100 000 copies/mL (5.0 log_10_). HIV RNA was undetectable in 10 PWH (48%) before switching to a regimen containing lenacapavir, fostemsavir, or both (Group A). These PWH had received multiple previous HIV treatment lines, with a mean of 18 regimens (range, 2–37). During the last regimen before switching, a mean of three active antiretroviral drugs remained available (range, 2–6), and eight PWH received at least four active drugs.

The number of previous virological failures was high in all three groups: median 12.5 (range, 1–23) in Group A, 12 (5–32) in Group B, and 19 (2–33) in Group C (Table 2). The difference among groups was not statistically significant (Kruskal–Wallis test, *p* = 0.62). Notably, one nonadherent PWH had never achieved virological suppression during their entire HIV treatment history (P21, who developed resistance to temsavir).

**TABLE 2:** Combination antiretroviral therapies in the 21 included PWH.

PWH	Antiretroviral therapy regimen	Group (A undetectable at baseline, B detectable with viral success, C detectable with persistent viral failure)	Genotypic Susceptibility Score (GSS) of the regimen containing fostemsavir and/or lenacapavir (number of drugs/therapeutic classes still active^a^)	Number of virological failures before fostemsavir and/or lenacapavir	Time to virological suppression in PWH viremic at baseline	Virological suppression maintenance (duration from virological suppression till end of follow‐up, or virological failure (VF))
1	Emtricitabine/Tenofovir Alafenamide/Bictegravir + Dolutegravir + **Fostemsavir**	A	1/2	13	—	12 months (no VF)
2	Emtricitabine/Tenofovir Disoproxil + **Lenacapavir** b	A	2.5/1	1	—	11 months (no VF)
3	Dolutegravir/Lamivudine + **Lenacapavir** b	A	1/1	23	—	6 months (no VF)
4	Cabotegravir + Doravirine + **Lenacapavir** b	B	12/3	6	1 month	12 months (no VF)
5	Dolutegravir/Lamivudine + **Fostemsavir + Lenacapavir**	A	4/1	12	—	VF at 17 months (92 then 108 copies/mL)
6	Lamivudine/Tenofovir Disoproxil/Doravirine + Maraviroc + Enfuvirtide + **Lenacapavir**	B	0/0	32	3 months	24 months (no VF)
7	Dolutegravir + Darunavir + Ritonavir + Ibalizumab + **Lenacapavir**	A	7/2	4	—	17 months (no VF)
8	Dolutegravir + Ritonavir + Lamivudine + Maraviroc + **Fostemsavir (+ Lenacapavir added 17 months later)**	B	0/0	19	10 months^c^	25 months (no VF)
9	Dolutegravir/Lamivudine + **Lenacapavir** b	A	7/2	20	—	18 months (no VF)
10	Dolutégravir + Maraviroc + Ritonavir + **Fostemsavir (+ Lenacapavir added 9 months later)**	B	0/1	5	15 months	13 months (no VF)
11	Dolutegravir + **Fostemsavir + Lenacapavir**	B	1/1	28	1 month	11 months (no VF)
12	**Fostemsavir + Lenacapavir**	**B**	14.5/3	10	2 months	NA^d^
13	Lamivudine/Tenofovir Disoproxil/Doravirine + **Fostemsavir**	B	3/2	14	6 months	14 months (no VF)
14	**Fostemsavir + Lenacapavir**	**B**	3/1	8	9 months	9 months (no VF)
15	Dolutegravir + Doravirine + **Lenacapavir**	A	7/1	15	—	12 months (no VF)
16	**Fostemsavir + Lenacapavir**	**A**	5/3	4	—	12 months (no VF)
17	Dolutegravir + Lamivudine + **Lenacapavir**	A	9/2	13	—	19 months (no VF)
18	Dolutegravir + **Fostemsavir** (+ **Lenacapavir** added 24 months later)	C	8.5/3	2	33 months^e^	14 months (no VF)
19	Dolutegravir + **Lenacapavir** b	A	1/1	6	—	13 months (VF)
20	Ibalizumab + **Fostemsavir + Lenacapavir**	C	1/1	33	1 month	VF at 4 months persisting till end of follow‐up (29 months later)
21	Emtricitabine/Rilpivirine/Tenofovir alafenamide + Doravirine + Enfuvirtide + **Fostemsavir (+ Lenacapavir added 10 months later)**	C	0/0	19	No viral suppression	VF from baseline to end of follow‐up (33 months later)

Abbreviations: PWH, people with HIV; NA, not available; VF, virological failure.

a Antiretroviral drug classes considered here were nucleos(t)ide reverse transcriptase inhibitors, non‐nucleos(t)ide reverse transcriptase inhibitors, protease inhibitors, and integrase inhibitors.

b Persons receiving only one daily oral tablet after switching, together with injections administered every 2 months for cabotegravir or every 6 months for lenacapavir.

c No viral load measurement was available between treatment initiation and the 10‐month visit.

d No follow‐up was available after 2 months.

e Virological suppression was achieved only after injectable cabotegravir was added to lenacapavir, 33 months after fostemsavir initiation.

The reason for switching to lenacapavir and/or fostemsavir was virological failure in 11 PWH, associated with immunological failure in seven, and other reasons in 10. We classified participants into three groups according to baseline viral load and subsequent virological evolution. Group A included 10 PWH with undetectable viral load at baseline. Reasons for switching in this group were treatment simplification in four PWH, tolerance issues or adverse effects in two, treatment intensification in two, and drug–drug interactions or pharmacological considerations in two (chemotherapy for lung cancer and tacrolimus, respectively). In this group, dyslipidemia was highly prevalent (7/10), and three PWH (P3, P9, and P16) were switched to lenacapavir and/or fostemsavir because metabolic syndrome with dyslipidemia contraindicated the initiation of a protease inhibitor. In one of these PWH, severe hypertriglyceridemia was observed (13.6 g/L, N < 1.5 g/L). After switching, four PWH required only one daily tablet combined with injections every 6 months (lenacapavir; P2, P3, P9, and P19; Table 2).

Median daily pill burden decreased and the number of injectable drugs (defined as the number of different molecules administered by injection) increased after switching in Group A, but not in the other groups (Supporting Information Figure S1). In Group A, the median number of pills decreased from 4 (range, 2–12) to 2 (range, 1–4) after switching (Wilcoxon test, *p* < 0.05). Overall, the number of antiretroviral molecules included in each regimen before and after switching did not differ significantly, either in the whole cohort or within each group.

Based on baseline HIV‐1 genotypic resistance testing, HIV‐1 showed resistance to at least two NRTIs in 20 PWH (95%), at least two NNRTIs in 20 (95%), at least two PIs in 13 (68%), and at least two INSTIs in 10 (63%). Four‐class drug‐resistant (4DR) HIV‐1 was identified in five PWH (24%). The mean genotypic susceptibility score (GSS) was 4.2 ± 4.2 (range, 0–14.5). GSS was ≥ 2 in 12 PWH and < 2 in nine PWH. Virological success with lenacapavir and/or fostemsavir was achieved in 11 of 12 PWH with GSS ≥ 2 and in eight of nine PWH with GSS < 2.

Adherence was assessed through review of medical records and consultation with the treating physicians. Overall, 11 PWH (52%) were considered adherent, eight (38%) nonadherent, and adherence could not be determined in two. Comparison of baseline characteristics across the three groups showed that the three PWH with persistent viremia (Group C) had lower CD4 nadirs and baseline CD4 counts, as well as higher baseline viral loads (Table 1). Notably, all three were identified as having poor treatment adherence both before and after switching to lenacapavir and/or fostemsavir.

### 3.2 Virological and Immunological Response to Combined Antiretroviral Treatment With Lenacapavir and/or Fostemsavir

**FIGURE 1:**
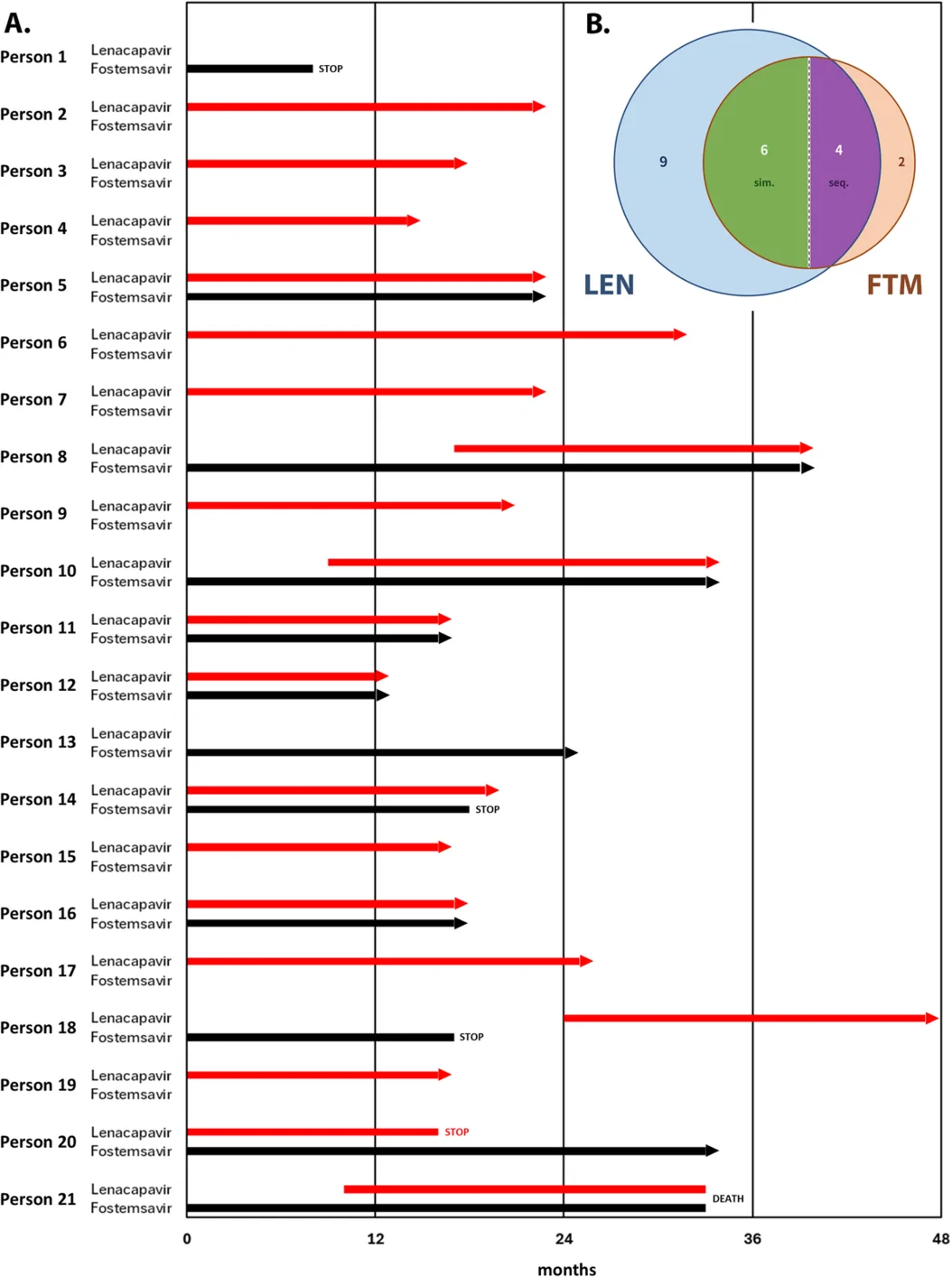
Treatment with lenacapavir and/or fostemsavir for the 21 included PWH. (A) Duration of treatment after inclusion. Arrows indicate that treatment was ongoing at the end of the authorized data collection period (July 2025). (B) Venn diagram representing the repartition of PWH with lenacapavir (LEN, *n* = 19) and/or fostemsavir (FTM, *n* = 12). Ten PWH had both drugs, including six with simultaneous initiation (sim.) and four with sequential initiation (FTM then LEN, seq.).

After treatment modification with incorporation of lenacapavir and/or fostemsavir, considering the date on which the first of these two agents was introduced, the cohort included nine PWH (43%) treated with lenacapavir without fostemsavir, six PWH (29%) receiving lenacapavir and fostemsavir simultaneously, and six PWH (29%) receiving fostemsavir without lenacapavir at baseline, including four in whom lenacapavir was added at least 6 months later and two who never received lenacapavir. No PWH received lenacapavir before fostemsavir (Figure 1A). Overall, 19/21 (90%) received lenacapavir and 12/21 (57%) received fostemsavir (Figure 1B). Table 2 summarizes the combined antiretroviral regimens.

Fostemsavir was administered to 12 PWH for a median duration of 20 months (range, 8–39 months). Treatment was discontinued in four PWH after 8, 17, 18, and 32 months. Reasons for discontinuation were prevention of hematological toxicity in one PWH receiving chemotherapy for cancer (P1), malaise with dizziness (P14), virological failure related to nonadherence (P18), and death (P21). In the remaining eight PWH, fostemsavir was ongoing at the end of the authorized data collection period in July 2025.

Lenacapavir was administered to 19 PWH for a median duration of 20 months (range, 12–31 months). Treatment was discontinued in two PWH after 16 and 23 months because of virological failure with resistance (P20) and death (P21). In the remaining 17 PWH, lenacapavir was ongoing at the end of the authorized data collection period in July 2025.

According to medical records, lenacapavir and fostemsavir were not associated with serious adverse events. One PWH (P21), who was hospitalized in a post‐acute care and rehabilitation center and monitored every 15 days for ibalizumab infusion and clinical assessment, died during follow‐up. This individual had acquired HIV through mother‐to‐child transmission, had documented poor adherence to antiretroviral therapy, and had immunovirological failure at treatment switch and during hospitalization before death, more than 2 years later. At the time of death, this PWH had received fostemsavir for more than 2 years and lenacapavir for more than 1 year and presented with influenza‐like symptoms leading to hospitalization and unexpected death. Death was therefore considered unrelated to treatment. Autopsy was proposed but declined by the family.

**FIGURE 2:**
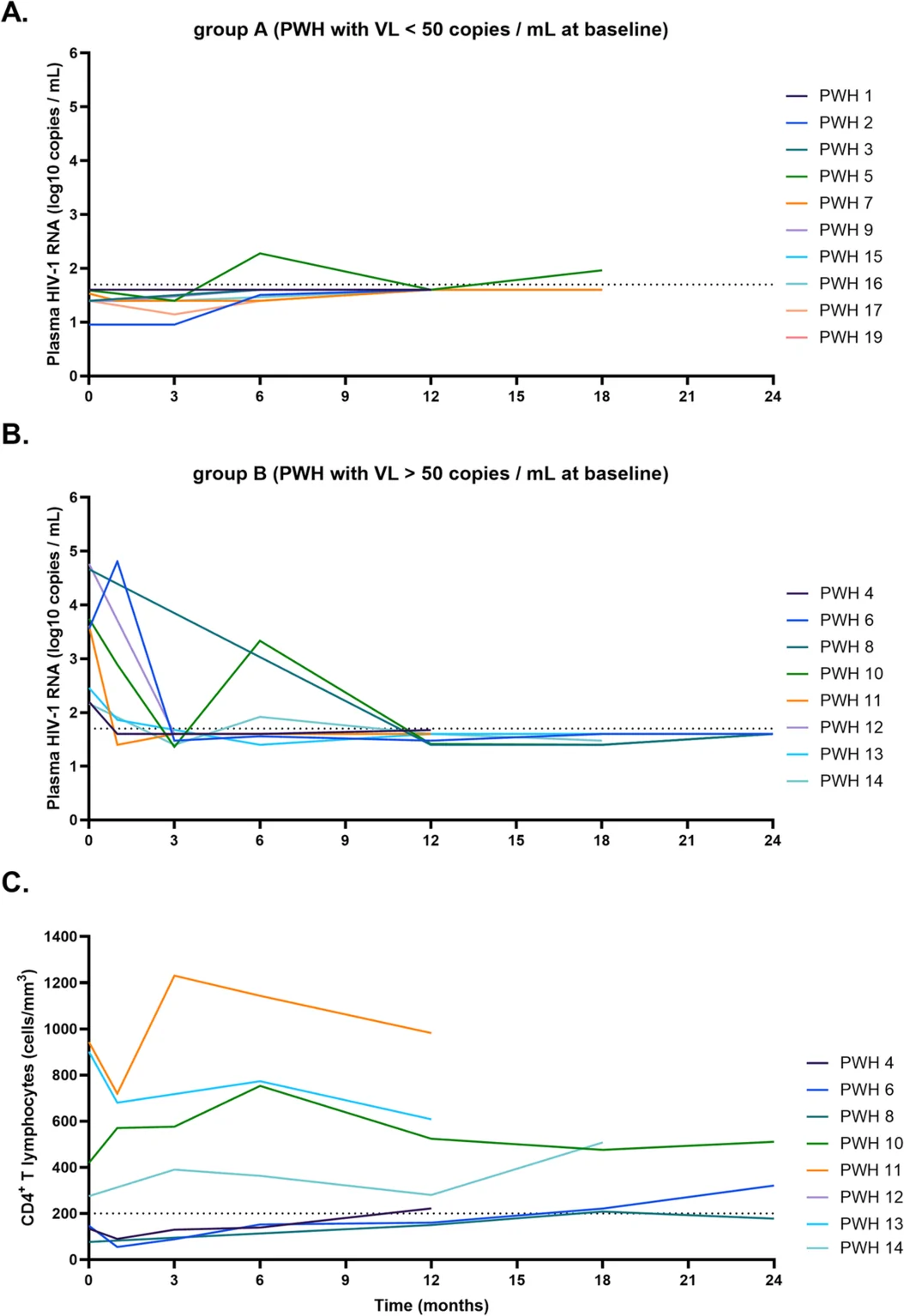
Longitudinal changes in HIV‐1 RNA load and CD4^+^ T‐cell counts from baseline to month 24. PWH: Person living with HIV, VL: Viral load. (A) Changes in viral load among PWH who were virologically suppressed at baseline (Group A). Changes in viral load (B) and CD4^+^ T‐cell counts (C) among PWH who were viremic at baseline and achieved viral suppression by month 24 (Group B). The three PWH with virological success and initial T CD4^+^ < 200 cells/mm^3^ at baseline increased their T CD4^+^ > 200 cells/mm^3^ after 12–18 months of treatment. The three PWH with persistent virological failure at month 24 are shown in Figure 3.

Among the 10 PWH with undetectable viral load at baseline (Group A), whose treatment switch was prompted by tolerance issues, drug–drug interactions, or treatment simplification, all but one maintained virological suppression without major adverse events (Figure 2A). One PWH experienced an isolated viral blip of 190 copies/mL at month 6, followed by viral suppression and then recurrent low‐level viremia at months 17 and 20 (< 200 copies/mL; P5), without immunological failure (CD4 count, 1016 cells/mm^3^).

**FIGURE 3:**
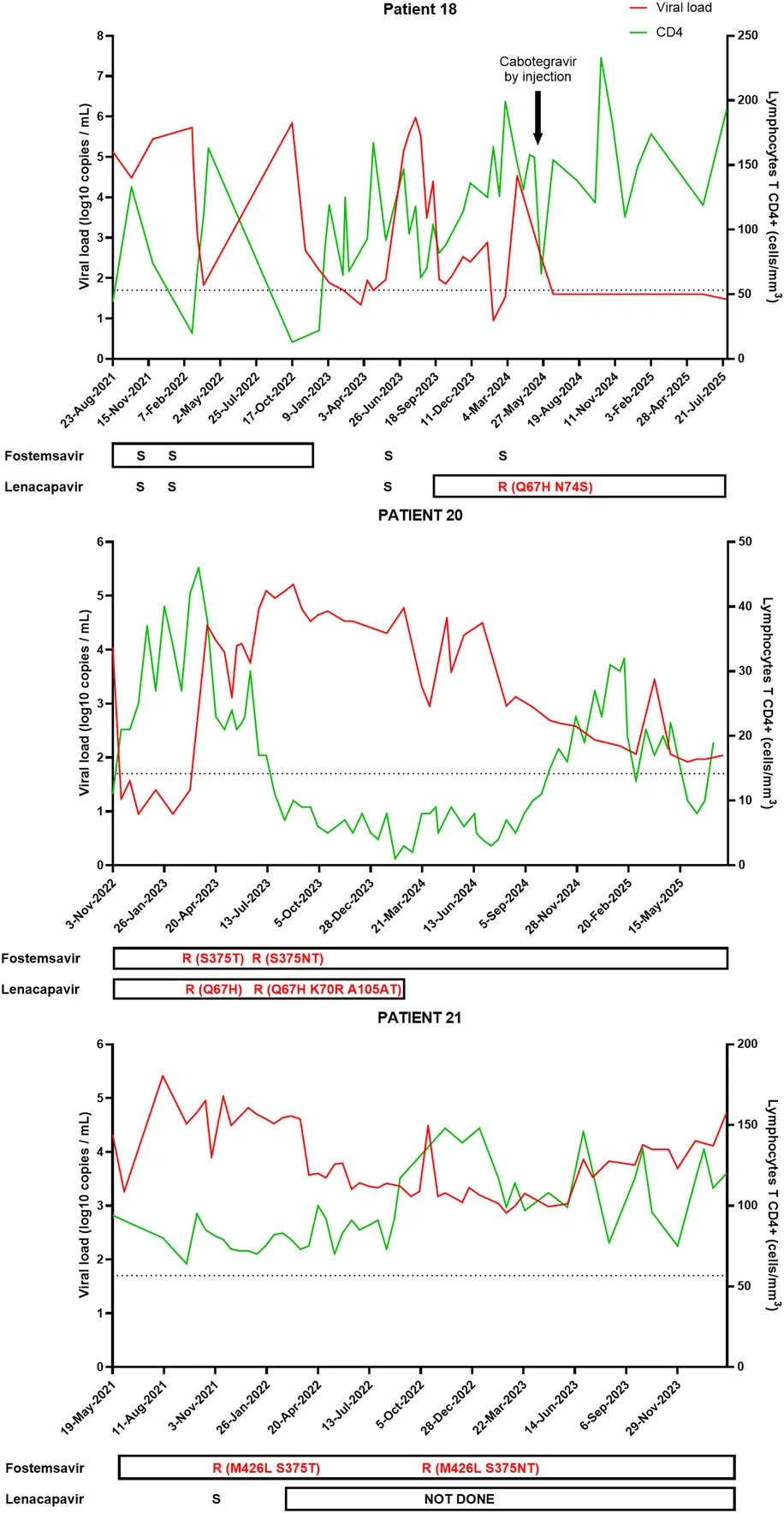
Detailed profiles of three PWH with persistent virological failure despite fostemsavir and/or lenacapavir treatment (Group C, *n* = 3). S, susceptible; R, resistant, with mutations conferring resistance. Boxes indicate treatment periods with the corresponding antiretroviral agent.

Among the 11 PWH with detectable viral load at baseline (Groups B and C), all except three (Group C: P18, P20, and P21) achieved viral suppression within 24 months after switching to the new regimens. After excluding P21, who never achieved viral suppression, the median time to viral suppression was 4.5 months (IQR, 1.0–11.25 months; range, 1–33 months; *n* = 10). Among the three PWH who had not achieved viral suppression at 24 months (Group C; Figure 3), P18, who was nonadherent, eventually achieved viral suppression at month 33 after switching to a regimen combining two injectable agents, cabotegravir and lenacapavir, with darunavir/ritonavir. P20 initially achieved viral suppression at month 1 but experienced rapid virological failure from month 4 until the end of follow‐up 29 months later. Finally, P21 never achieved viral suppression, as noted above. Among the eight PWH who achieved viral suppression by 24 months (Group B), all maintained viral suppression until the end of follow‐up without virological relapse (median duration of viral suppression, 13.5 months; range, 9–25 months). Overall, eight of 11 PWH (72.7%) with detectable viral load at baseline achieved sustained viral suppression at 12 and 24 months (Figure 2B), and nine of 11 (81.8%) had achieved viral suppression by 36 months, after P18 achieved suppression at month 33.

To better characterize immunological outcomes, we analyzed changes in CD4 cell counts under the new regimens. Overall, no significant increase in CD4 count was observed after switching. Among the 10 PWH with undetectable viral load at baseline, seven maintained CD4 counts > 200 cells/mm^3^ during treatment, one had CD4 counts < 200 cells/mm^3^ before and after treatment, one increased from < 200 to > 200 cells/mm^3^ after treatment, and one decreased from > 200 to < 200 cells/mm^3^ after treatment (187 cells/mm^3^). Among the 11 PWH with detectable viral load at baseline, five maintained CD4 counts > 200 cells/mm^3^, and three increased from < 200 to > 200 cells/mm^3^ (Figure 2C). Only in this last group of three PWH with < 200 cells and virological suppression at 24 months, CD4^+^ T‐cells counts increased significantly over time after treatment initiation (linear regression, +7.9 cells per month, one‐sided *t*‐test, *p* = 0.044; first‐to‐last‐change +173 cells, *p* < 0.001). The three PWH who remained below 200 cells/mm^3^ (immunological failure) were the same three nonadherent PWH with persistent detectable viremia and documented resistance (Group C: P18, P20, and P21; Figure 3). Notably, all PWH with isolated virological failure at baseline, without concomitant immunological failure, achieved undetectable viral load (4/4). Among the seven PWH with immunovirological failure at baseline, only three had persistent virological failure, which was associated with poor adherence and the emergence of resistance to the newly introduced drugs (Group C).

### 3.3 Genotypic Drug Resistance to Lenacapavir and Fostemsavir

Sixteen *gag* and 12 *env* sequences suitable for interpretation of genotypic resistance to lenacapavir and fostemsavir, respectively, were obtained from 12 and eight PWH, respectively (Supporting Information Figure S2). HIV‐1 subtypes determined from *gag* and *env* sequences were concordant with those determined from reverse transcriptase sequences. Before initiation of lenacapavir and/or fostemsavir, 11 *gag* and eight *env* sequences were obtained from nine and seven PWH, respectively. Both *gag* and *env* sequences were available for six PWH. In one PWH, sequences were obtained from two different plasma samples. These HIV‐1 sequences were obtained a mean of 12 months before drug introduction (range, 0–18 months). Fourteen sequences were obtained from plasma and five from peripheral blood mononuclear cells. HIV‐1 was susceptible to lenacapavir in all PWH with available pretreatment sequences. HIV‐1 was also susceptible to fostemsavir in all PWH with available pretreatment sequences, except for one PWH whose virus already harbored an S375I/S substitution in *env*, conferring possible resistance to fostemsavir.

After initiation of lenacapavir and/or fostemsavir, five *gag* and four *env* sequences were obtained from three and two PWH, respectively, for assessment of lenacapavir and fostemsavir resistance. Post‐treatment sequences were obtained a mean of 6 months after treatment initiation (range, 4–12 months). Eight sequences were obtained from plasma and one from peripheral blood mononuclear cells. Emergence of resistance was identified only in three PWH with documented nonadherence and persistent virological failure (Group C; detailed below).

### 3.4 Plasma Antiretroviral Concentrations in Groups A and B

**FIGURE 4:**
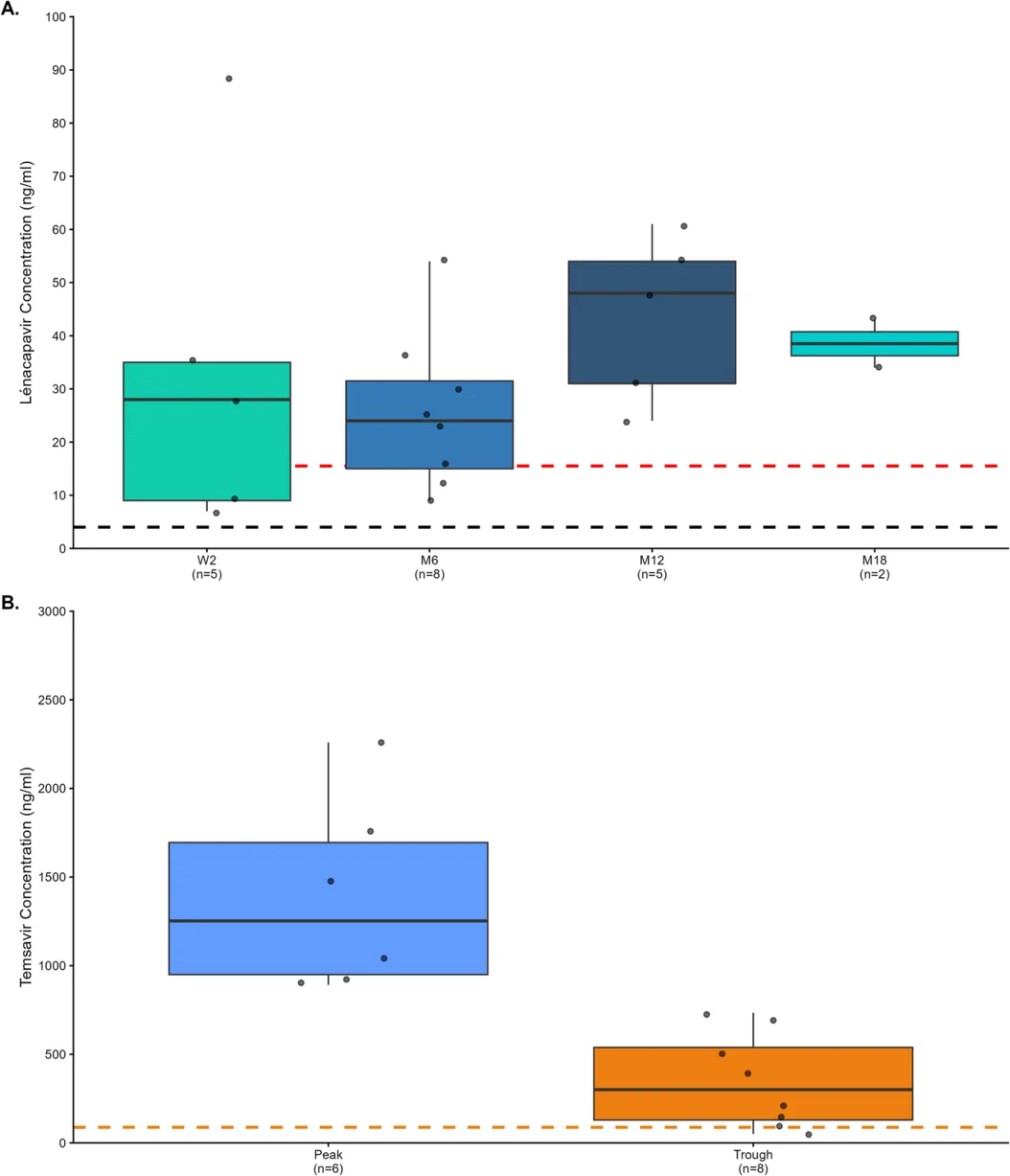
Plasma concentrations of lenacapavir and temsavir during follow‐up. (A) Box plots of lenacapavir trough concentrations. The dashed black line represents the lower limit of the therapeutic range, and the dashed red line represents the four‐fold protein‐adjusted IC95 threshold (15.5 ng/mL). (B) Box plots of temsavir peak concentrations (blue) and trough concentrations (orange). The dashed orange line represents the lower limit of the therapeutic range. Box boundaries indicate the 25th and 75th percentiles, the line within each box indicates the median, and whiskers indicate the fifth and 95th percentiles. Black dots represent outliers.

We report here plasma drug concentrations for Groups A (virologically suppressed at baseline) and B (viremic at baseline but achieving viral suppression by 24 months). Plasma concentrations for the three PWH in Group C (persistent viremia at 24 months) are presented separately in the section “Detailed analysis of three persons developing high‐level resistance and persistent viral failure.” During follow‐up, drug concentrations were available for 12 of 16 PWH treated with lenacapavir and for nine of 12 PWH treated with fostemsavir in Groups A and B, corresponding to 20 lenacapavir and 14 temsavir samples, respectively. Median (range; coefficient of variation [%]; *n*) lenacapavir trough concentrations were 28 ng/mL (7–88 ng/mL; 98.2%; *n* = 5) at the end of the oral lead‐in phase (week 2), 24 ng/mL (9–54 ng/mL; 57%; *n* = 8) at month 6, 48 ng/mL (24–61 ng/mL; 35.7%; *n* = 5) at month 12, and 38.5 ng/mL (34–43 ng/mL; 16.5%; *n* = 2) at month 18 (Figure 4). At the end of the oral lead‐in phase, TDM was performed in only four PWH (P2, P11, P14, and P16). Two of them (P2 and P16) had low lenacapavir concentrations (9 and 7 ng/mL, respectively), below the recommended threshold of 15.5 ng/mL, probably because of poor adherence or reduced oral bioavailability. After the first subcutaneous injection, lenacapavir concentration in P2 remained below the recommended threshold (9 ng/mL) but increased after the second injection (31 ng/mL). Another PWH (P7) had a lenacapavir Ctrough slightly below the recommended threshold after the first injection (12 ng/mL), but the concentration reached target levels 3 months after the second injection and remained stable thereafter.

Median (range; coefficient of variation [%]; *n*) temsavir peak and trough concentrations, measured at steady state at least 1 month after initiation, were 1252 ng/mL (891–2260 ng/mL; 39.4%; *n* = 6) and 300 ng/mL (< 50–754 ng/mL; 77%; *n* = 8), respectively (Figure 4). Only one PWH (P16) had an undetectable Ctrough (< 50 ng/mL) during follow‐up.

TDM of concomitant oral antiretroviral drugs was performed in parallel in 15 of 18 PWH. Drug concentrations were within the therapeutic ranges in 11 PWH but were below the therapeutic ranges on several occasions in four PWH (P2, P5, P7, and P13).

### 3.5 Detailed Analysis of Three PWH Developing High‐Level Resistance and Persistent Virological Failure

In three PWH with previously documented nonadherence (Group C: P18, P20, and P21, Figure 3), virological failure persisted and was consistent with suspected ongoing nonadherence, variable plasma concentrations of oral antiretroviral therapy, and emergence of resistance to lenacapavir in P18 and P20 and to fostemsavir in P20 and P21 (Figure 4). The baseline GSS values for P18, P20, and P21 were 8.5, 1, and 0, respectively.

For P18, sequences obtained at two time points before switching and at 1, 5, and 22 months after treatment modification showed no resistance mutations. This PWH initiated fostemsavir in September 2021, discontinued it in February 2023, and started lenacapavir in February 2023. During follow‐up after the treatment switch, 22 blood samples were analyzed. Temsavir concentrations were undetectable at months 3, 6, and 13 after fostemsavir initiation, whereas they were within the therapeutic range at months 1, 7, 17, and 19, indicating repeated nonadherence. None of the four sequences obtained at 1, 5, 22, and 30 months after starting fostemsavir showed mutations conferring resistance, although viral loads were 4.5, 5.7, 6.0, and 4.0 log10 copies/mL, respectively, consistent with nonadherence without selection of fostemsavir resistance. For lenacapavir, concentrations measured during follow‐up (49 ng/mL at month 4, 29 ng/mL at month 6, 38 ng/mL at month 12, 229 ng/mL at month 14, and 188 ng/mL at month 15) were within the therapeutic range and above the recommended threshold of 15.5 ng/mL, indicating adequate adherence to injections. In contrast, undetectable plasma dolutegravir concentrations were observed on four occasions while dolutegravir was administered concomitantly with fostemsavir and subsequently with lenacapavir. Eight months after lenacapavir initiation, the Q67H and N74S mutations, which confer resistance to lenacapavir, were detected by RNA sequencing. Dolutegravir was then replaced by injectable cabotegravir, and oral darunavir/ritonavir was added to injectable lenacapavir. This regimen resulted in undetectable viral load 1 month after the switch, with viral suppression maintained until the end of follow‐up 23 months later (Figure 3). Cabotegravir and darunavir trough concentrations remained within the therapeutic range at each visit.

For P20, who was treated with lenacapavir and fostemsavir, *gag* and *env* sequences were obtained 6 and 10 months after introduction of these drugs. Regarding lenacapavir, HIV‐1 harboring the gag mutation Q67H was detected at month 6, conferring resistance according to the ANRS‐MIE algorithm and intermediate resistance according to the Stanford algorithm. At month 10, HIV‐1 gag harbored the K70R and A105AT mutations in addition to Q67H. Regarding fostemsavir, HIV‐1 harbored the *env* mutation S375T in both post‐treatment samples, conferring possible resistance. Nine blood samples with therapeutic drug monitoring were obtained, showing highly variable temsavir concentrations and low concentrations of oral concomitant antiretrovirals, including four of five dolutegravir concentrations below therapeutic levels, supporting nonadherence to treatment. Lenacapavir quantification was not available at the time of treatment, before 2024.

For PWH 21, temsavir concentrations were undetectable at months 13, 15, and 31 but within the therapeutic range at months 8 and 17, whereas doravirine concentrations were consistently within the therapeutic range. HIV‐1 sequences harbored the M426L and S375T mutations conferring resistance to fostemsavir at 6 and 16 months after initiation. None of the sequences obtained before treatment or 6 months after initiation showed mutations conferring resistance to lenacapavir. Assessment of lenacapavir plasma concentrations and resistance‐associated mutations could not be performed on the sample collected 16 months after initiation. Temsavir was maintained because no alternative options were available.

## 4 Discussion

This real‐world study provides clinically relevant data on lenacapavir and fostemsavir in 21 people with HIV (PWH) and multidrug‐resistant HIV‐1 who had been living with HIV for approximately three decades. Most participants were heavily treatment‐experienced, with up to 37 previous antiretroviral regimens. Although the efficacy and safety of these agents have been demonstrated in clinical trials [8, 16], data from real‐world context are just beginning to emerge with cases reports since 2023 [23, 24] and cohort studies since 2025.

In 2025, in a U.S. cohort of 21 PWH treated with LEN plus ibalizumab, the overall virological success rate was 66% among participants who were either suppressed or viremic at baseline [9]. An Italian study reported 15 PWH with multidrug‐resistant HIV, including eight who also received fostemsavir; 11 of 15 (73%) achieved virological suppression within 3 months, whereas LEN resistance mutations were detected in the only participant with persistent viremia [13]. In 2026 in France, Delaugerre et al. reported viral suppression at week 26 in 86% of participants already suppressed and in 53% of those viremic at baseline, with emergence of Q67H in one case [11]. Charpentier et al. reported viral suppression rates of 94% among participants suppressed and 64% among those viremic at baseline, with emerging N74D resistance [12]. More recently, Kaperak et al. reported virological success in 89% of 38 viremic PWH in a multicenter U.S. real‐world cohort [10]. In our study, overall viral suppression was achieved in 19 of 21 PWH (90.5%), including nine of 10 (90%) who were already suppressed at baseline and nine of 11 (81.8%) who were viremic at baseline. This rate compares favorably with most previous real‐world studies and is close to the most recent multicenter U.S. data. In the OPERA study, which assessed virological response to FTM in 116 persons with detectable HIV viral load at baseline, 22% achieved undetectable HIV RNA after 12 months [25]. Thus, globally, LEN seems to be associated both in clinical trials and observational evidence with better virological success than FTM for HTE PWH. In our study, comparison of LEN and FTM was not possible since 19/21 PWH had LEN, including 10 with FTM (six with simultaneous initiation and four with delayed initiation, Figure 1B). Strikingly, 2/3 PWH with persistent virological failure (group C) had delayed LEN and failed first with FTM then failed again when LEN was introduced 10 (P21) and 24 (P18) months after FTM. This suggests that, in our study, nonadherence was the strongest predictor for virological failure regardless of LEN or FTM treatment.

In our cohort, the three cases of persistent virological failure occurred in nonadherent PWH with baseline immunological failure, lower CD4 nadirs, lower baseline CD4 counts, and higher baseline viral loads. These results will help identifying the people at risk of viral failure despite switching to these new drugs and requiring more careful monitoring. These three PWH developed resistance to lenacapavir in two cases and to fostemsavir in two cases, including one PWH who developed resistance to both drugs (P20; Figure 3).

Resistance emergence in our cohort also reinforces observations from CAPELLA, in which capsid resistance mutations such as M66I and Q67H were mainly observed in participants with poor adherence or functional monotherapy. In the PWH with isolated lenacapavir resistance in our cohort (P18), replacing oral dolutegravir with injectable cabotegravir, while continuing injectable lenacapavir and adding boosted darunavir, restored and maintained viral suppression. This case highlights the potential value of dual‐injectable rescue strategies in selected PWH with multidrug‐resistant HIV and major adherence challenges.

Focusing on virologically suppressed people living with HIV (PWH), FTM, and/or LEN have provided solutions in complex situations not due to immunovirological factors, but rather when standard drug classes were contraindicated due to drug interactions or patient‐specific factors. For example, it is worth noting that dyslipidemia was very common among these patients (70%), with one case of severe hypertriglyceridemia (P3), possibly related to the duration of infection and a history of treatment with boosted protease inhibitors. These two drugs therefore represent treatment options for PWH with metabolic syndrome for whom boosted PIs could not be used.

Lenacapavir and fostemsavir were generally well tolerated. No serious adverse event was attributed to either drug. Fostemsavir was discontinued in one PWH because of malaise and dizziness, although causality remained uncertain. Injection‐site reactions were consistent with the known safety profile of lenacapavir and were not severe. These findings are in line with CAPELLA [10, 26] and BRIGHTE [16], in which severe drug‐related adverse events were uncommon, and injection‐site reactions were usually mild or moderate.

Therapeutic drug monitoring provided additional insight into treatment exposure and adherence. High interindividual variability was observed for temsavir and during the oral lead‐in phase of lenacapavir, whereas variability decreased after subcutaneous lenacapavir administration. In PWH who achieved viral suppression (Groups A and B), lenacapavir and temsavir concentrations were generally within recommended therapeutic ranges. However, subtherapeutic concentrations of concomitant oral antiretrovirals were observed in approximately one quarter of monitored PWH, emphasizing the importance of adherence to the optimized background regimen. Our findings support previous observations that inadequate adherence to companion oral therapy may expose PWH to functional lenacapavir monotherapy and facilitate resistance selection [26, 27, 28]. In selected nonadherent PWH, early consideration of combinations, including two injectable agents may reduce the risk of functional monotherapy and resistance emergence.

A key limitation of our study is that adherence was not assessed systematically with standardized tools; clinical assessment alone may fail to capture this major determinant of therapeutic and virological outcomes [29]. Despite the limited sample size, this study addresses a timely clinical question and provides observational data independent of clinical trials. The cohort size is comparable to several recent real‐world studies, including those by Rolle [11], Clemente [15], and Delaugerre [13], although smaller than the larger multicenter cohorts reported by Kaperak [12] and Charpentier [14]. Its main strength lies in the detailed clinical, virological, resistance, and pharmacological characterization of a complex population treated in routine care.

Another strength is the early access to these drugs in our center. Fostemsavir was first prescribed in May 2021, shortly after European approval, and lenacapavir was first prescribed in January 2023, soon after its European market authorization. Consequently, follow‐up was relatively long for a real‐world cohort, with 19 PWH having at least 12 months of follow‐up and 10 having at least 24 months. Few real‐world studies have yet reported such longitudinal experience with these agents.

In summary, lenacapavir and fostemsavir were effective and well tolerated in this small real‐world cohort of PWH with multidrug‐resistant HIV‐1. High rates of viral suppression were achieved, including among initially viremic PWH, and no serious drug‐related adverse events were observed. However, persistent virological failure and resistance emergence occurred in nonadherent PWH with advanced treatment histories, underscoring the critical importance of adherence support, therapeutic drug monitoring, and careful optimization of companion antiretroviral drugs. In selected complex cases, dual‐injectable rescue strategies, including lenacapavir and cabotegravir may represent an important therapeutic option. Larger multicenter real‐world studies with standardized adherence assessment and longer follow‐up are needed to confirm these findings.

## Author Contributions

Conceptualization: Philippe Colson and Matthieu Million. Methodology: Syrine Boujamline, Celine Boschi, Nadege Neant, Caroline Solas, Philippe Colson, and Matthieu Million. Resources and clinical data collection: all authors. Formal analysis: Syrine Boujamline, Celine Boschi, Nadege Neant, Caroline Solas, Philippe Colson, and Matthieu Million. Writing – original draft preparation: Syrine Boujamline, Caroline Solas, Philippe Colson, and Matthieu Million. Writing – review and editing: all authors. All authors have read and approved the final version of the manuscript.

## Ethics Statement

The present study involves data registered on the Health Data Access Portal of Marseille University and public hospitals (Assistance Publique‐Hôpitaux de Marseille, AP‐HM) under No. PADS25‐338 and was approved by the AP‐HM Ethics and Scientific Committee under No. CSE25‐343.

## Conflicts of Interest

Syrine Boujamline, Celine Boschi, Saadia Mokhtari, Yacine Belkhir, Nadege Neant, and the remaining authors not listed below declare no conflicts of interest. Hugo Bes Barlandier has received support from MSD, Gilead, and ViiV Healthcare. Christelle Tomei has received support from MSD. Amelie Menard has received support from MSD, Gilead, ViiV Healthcare, and GSK. Isabelle Ravaux has received support from MSD, Gilead, ViiV Healthcare, and AbbVie. Caroline Solas has received support from MSD, Gilead, ViiV Healthcare, and Pfizer. Philippe Colson serves as a scientific advisor for BioSellal and Triber. Matthieu Million has received support from GSK. The funding sources had no role in the study design; data collection, management, analysis, or interpretation; manuscript preparation, review, or approval; or the decision to submit the manuscript for publication.

## Policy on Using ChatGPT and Similar AI Tools

Microsoft Copilot was used solely for language editing and rephrasing to improve clarity, fluency, and scientific style. It was not used for data generation, data analysis, interpretation of results, literature selection, or scientific decision‐making.

## Supporting information


Supporting File 1

Supporting File 2

Supporting File 3

## Data Availability

The data that support the findings of this study are available from the corresponding author upon reasonable request.
