# Supplementary material for: Real‐World Experience With Lenacapavir and/or Fostemsavir as Emerging Therapies for the Management of Multidrug‐Resistant HIV‐1 in Southeastern France
Source: J Med Virol. 2026 Sep 11;98(9):e71149. doi: 10.1002/jmv.71149 (PMC13563682; doi:10.1002/jmv.71149)
Supplement: Supplementary file 1 [file JMV-98-e71149-s001.pdf]

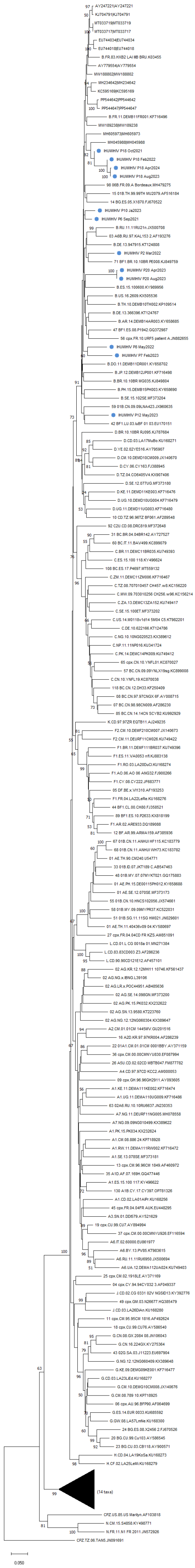
(14 taxa)
0.050
