# Supplementary material for: Real‐World Experience With Lenacapavir and/or Fostemsavir as Emerging Therapies for the Management of Multidrug‐Resistant HIV‐1 in Southeastern France
Source: J Med Virol. 2026 Sep 11;98(9):e71149. doi: 10.1002/jmv.71149 (PMC13563682; doi:10.1002/jmv.71149)
Supplement: Supplementary file 2 [file JMV-98-e71149-s002.pdf]

Real-World Experience with Lenacapavir and/or Fostemsavir as Emerging Therapies for the Management of Multidrug-Resistant HIV-1 in Southeastern France

Syrine BOUJAMLINE, Celine BOSCHI, Hugo BES BARLANDIER, Christelle TOMEI, Amelie MENARD, Saadia MOKHTARI, Isabelle RAVAUX, Yacine BELKHIR, Nadege NEANT, Caroline SOLAS, Philippe COLSON, Matthieu MILLION

SUPPLEMENTARY MATERIAL

Supplementary Figure S1. Number of pills, injectable drugs and molecules before and after switch to lenacapavir and/or fostemsavir

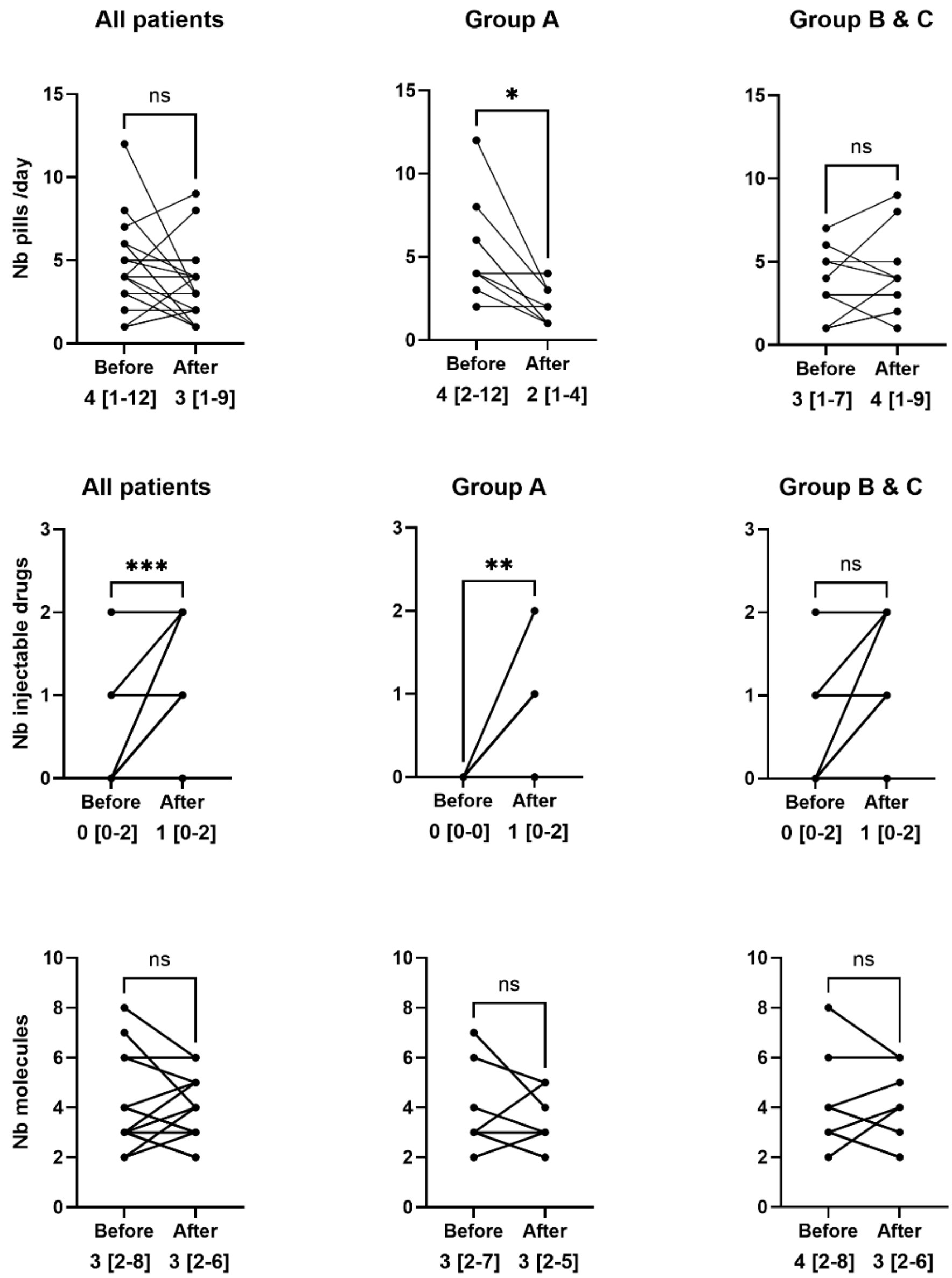

Group A : PWH with undetectable VL at baseline (n = 10), Group B & C (n = 11) : PWH with detectable viral load at baseline and viral suppression (Group B, n = 8) or persistent viral failure (Group C, n = 3). Numbers below graphs are medians and ranges. Number of injectable drugs represent the number of different injectable molecules used.

Supplementary Figure S2. Phylogenetic analyses were performed based on HIV-1 *gag* (A) and *env* (B) sequences

The HIV-1 *gag* and *env* sequences generated in this study were submitted to the GenBank sequence database and assigned accession numbers PV899489–PV899516. Classification of HIV-1 sequences was performed using BLAST (1) and HIV BLAST web applications (https://www.hiv.lanl.gov/content/sequence/BASIC_BLAST/basic_blast.html). Reference HIV-1 sequences used for *gag* and *env* were obtained from the Los Alamos National Library HIV sequence database (2). For phylogenetic reconstruction, nucleotides alignments were performed using the MAFFT web application (https://mafft.cbrc.jp/alignment/server/index.html) (3). Evolutionary analyses were conducted in MEGA11 (4). The evolutionary history was inferred using the Neighbor-Joining method. The evolutionary distances were computed using the Kimura 2-parameter method and are in the units of the number of base substitutions per site. The percentage of replicate trees in which the associated taxa clustered together in the bootstrap test (1000 replicates) were shown next to the branches. The tree was drawn to scale, with branch lengths in the same units as those of the evolutionary distances used to infer the phylogenetic tree.

(A) Analysis involved 195 *gag* nucleotides sequences. There were a total of 1951 positions for lenacapavir in the final dataset. Sequences marked with a blue circle belong to the patients in this study.

(B) Analysis involved 190 *env* nucleotide sequences. There were a total of 1054 positions in the final dataset. Sequences marked with a blue circle belong to the patients in this study.

REFERENCES

Altschul SF, Gish W, Miller W, Myers EW, Lipman DJ. Basic local alignment search tool. J Mol Biol. 1990 Oct 5;215(3):403-10. doi: 10.1016/S0022-2836(05)80360-2. PMID: 2231712.

Kuiken C, Korber B, Shafer RW. HIV sequence databases. AIDS Rev. 2003 Jan-Mar;5(1):52-61. PMID: 12875108; PMCID: PMC2613779.

Katoh K, Rozewicki J, Yamada KD. MAFFT online service: multiple sequence alignment, interactive sequence choice and visualization. Brief Bioinform. 2019 Jul 19;20(4):1160-1166. doi: 10.1093/bib/bbx108. PMID: 28968734; PMCID: PMC6781576.

Tamura K, Stecher G, Kumar S. MEGA11: Molecular Evolutionary Genetics Analysis Version 11. Mol Biol Evol. 2021 Jun 25;38(7):3022-3027. doi: 10.1093/molbev/msab120. PMID: 33892491; PMCID: PMC8233496.
