# Supplementary material for: Real‐World Experience With Lenacapavir and/or Fostemsavir as Emerging Therapies for the Management of Multidrug‐Resistant HIV‐1 in Southeastern France
Source: J Med Virol. 2026 Sep 11;98(9):e71149. doi: 10.1002/jmv.71149 (PMC13563682; doi:10.1002/jmv.71149)
Supplement: Supplementary file 3 [file JMV-98-e71149-s003.pdf]

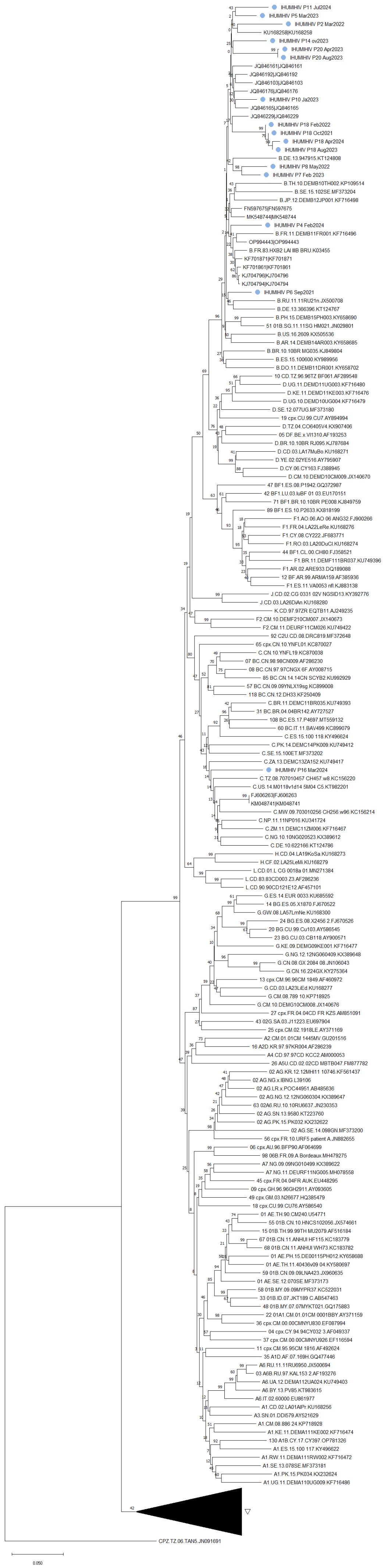
IHUMIHIV P11 Jul2024
IHUMIHIV P5 Mar2023
IHUMIHIV P2 Mar2022
KU168258|KU168258
IHUMIHIV P14 ov2023
IHUMIHIV P20 Apr2023
IHUMIHIV P20 Aug2023
JQ846161|JQ846161
JQ846192|JQ846192
JQ846103|JQ846103
JQ846176|JQ846176
IHUMIHIV P10 Ja2023
JQ846165|JQ846165
JQ846229|JQ846229
IHUMIHIV P18 Feb2022
IHUMIHIV P18 Oct2021
IHUMIHIV P18 Apr2024
IHUMIHIV P18 Aug2023
B.DE.13.947915.KT124808
IHUMIHIV P8 May2022
IHUMIHIV P7 Feb 2023
B.TH.10.DEMB10TH002.KP109514
B.SE.15.102SE.MF373204
B.JP.12.DEMB12JP001.KF716498
FN597675|FN597675
MK548744|MK548744
IHUMIHIV P4 Feb2024
B.FR.11.DEMB11FR001.KF716496
OP994443|OP994443
B.FR.83.HXB2 LAI IIIB BRU.K03455
KF701871|KF701871
KF701861|KF701861
KJ704796|KJ704796
KJ704794|KJ704794
IHUMIHIV P6 Sep2021
B.RU.11.11RU21n.JX500708
B.DE.13.366396.KT124767
B.PH.15.DEMB15PH003.KY658690
51 01B.SG.11.11SG HM021.JN029801
B.US.16.2609.KX505536
B.AR.14.DEMB14AR003.KY658685
B.BR.10.10BR MG035.KJ849804
B.ES.15.100600.KY989956
B.DO.11.DEMB11DR001.KY658702
10 CD.TZ.96.96TZ BF061.AF289548
D.UG.11.DEMD11UG003.KF716480
D.KE.11.DEMD11KE003.KF716476
D.UG.10.DEMD10UG004.KF716479
D.SE.12.077UG.MF373180
19 cpx.CU.99.CU7.AY894994
D.TZ.04.CO6405V4.KX907406
05 DF.BE.x.VI1310.AF193253
D.BR.10.10BR RJ095.KJ787684
D.CD.03.LA17MuBo.KU168271
D.YE.02.02YE516.AY795907
D.CY.06.CY163.FJ388945
D.CM.10.DEMD10CM009.JX140670
47 BF1.ES.08.P1942.GQ372987
42 BF1.LU.03.luBF 01 03.EU170151
71 BF1.BR.10.10BR PE008.KJ849759
89 BF1.ES.10.P2633.KX818199
F1.AO.06.AO 06 ANG32.FJ900266
F1.FR.04.LA22LeRe.KU168276
F1.CY.08.CY222.JF683771
F1.RO.03.LA20DuCl.KU168274
44 BF1.CL.00.CH80.FJ358521
F1.BR.11.DEMF111BR037.KU749396
F1.AR.02.ARE933.DQ189088
12 BF.AR.99.ARMA159.AF385936
F1.ES.11.VA0053 nfl.KJ883138
J.CD.02.CG 0331 02V NGSID13.KY392776
J.CD.03.LA26DiAn.KU168280
K.CD.97.97ZR EQTB11.AJ249235
F2.CM.10.DEMF210CM007.JX140673
F2.CM.11.DEURF11CM026.KU749422
92 C2U.CD.08.DRC819.MF372648
65 cpx.CN.10.YNFL01.KC870027
C.CN.10.YNFL19.KC870038
07 BC.CN.98.98CN009.AF286230
08 BC.CN.97.97CNGX 6F.AY008715
85 BC.CN.14.14CN SCYB2.KU992929
57 BC.CN.09.09YNLX19sg.KC899008
118 BC.CN.12.DH33.KF250409
C.BR.11.DEMC11BR035.KU749393
31 BC.BR.04.04BR142.AY727527
108 BC.ES.17.P4697.MT559132
60 BC.IT.11.BAV499.KC899079
C.ES.15.100 118.KY496624
C.PK.14.DEMC14PK009.KU749412
C.SE.15.100ET.MF373202
C.ZA.13.DEMC13ZA152.KU749417
IHUMIHIV P16 Mar2024
C.TZ.08.707010457 CH457.w8.KC156220
C.US.14.M0118v1d14 5M04 C5.KT982201
FJ606263|FJ606263
KM048741|KM048741
C.MW.09.703010256 CH256.w96.KC156214
C.NP.11.11NP016.KU341724
C.ZM.11.DEMC11ZM006.KF716467
C.NG.10.10NG020523.KX389612
C.DE.10.622166.KT124786
H.CD.04.LA19KoSa.KU168273
H.CF.02.LA25LeMi.KU168279
L.CD.01.L CG 0018a 01.MN271384
L.CD.83.83CD003 Z3.AF286236
L.CD.90.90CD121E12.AF457101
G.ES.14.EUR 0033.KU685592
14 BG.ES.05.X1870.FJ670522
G.GW.08.LA57LmNe.KU168300
24 BG.ES.08.X2456 2.FJ670526
20 BG.CU.99.Cu103.AY586545
23 BG.CU.03.CB118.AY900571
G.KE.09.DEMG09KE001.KF716477
G.NG.12.12NG060409.KX389648
G.CN.08.GX 2084 08.JN106043
G.CN.16.224GX.KY275364
13 cpx.CM.96.96CM 1849.AF460972
G.CD.03.LA23LiEd.KU168277
G.CM.08.789 10.KP718925
G.CM.10.DEMG10CM008.JX140676
27 cpx.FR.04.04CD FR KZS.AM851091
43 02G.SA.03.J11223.EU697904
25 cpx.CM.02.1918LE.AY371169
A2.CM.01.01CM 1445MV.GU201516
16 A2D.KR.97.97KR004.AF286239
A4.CD.97.97CD KCC2.AM000053
26 A5U.CD.02.02CD MBTB047.FM877782
02 AG.KR.12.12MHI11 10746.KF561437
02 AG.NG.x.IBNG.L39106
02 AG.LR.x.POC44951.AB485636
02 AG.NG.12.12NG060304.KX389647
63 02A6.RU.10.10RU6637.JN230353
02 AG.SN.13.9580.KT223760
02 AG.PK.15.PK032.KX232622
02 AG.SE.14.098GN.MF373200
56 cpx.FR.10.URF5 patient A.JN882655
06 cpx.AU.96.BFP90.AF064699
98 06B.FR.09.A Bordeaux.MH479275
A7.NG.09.09NG010499.KX389622
A7.NG.11.DEURF11NG005.MH078558
45 cpx.FR.04.04FR AUK.EU448295
09 cpx.GH.96.96GH2911.AY093605
49 cpx.GM.03.N26677.HQ385479
18 cpx.CU.99.CU76.AY586540
01 AE.TH.90.CM240.U54771
55 01B.CN.10.HNCS102056.JX574661
15 01B.TH.99.99TH MU2079.AF516184
67 01B.CN.11.ANHUI HF115.KC183779
68 01B.CN.11.ANHUI WH73.KC183782
01 AE.PH.15.DE00115PH012.KY658688
01 AE.TH.11.40436v09 04.KY580697
59 01B.CN.09.09LNA423.JX960635
01 AE.SE.12.070SE.MF373173
58 01B.MY.09.09MYPR37.KC522031
33 01B.ID.07.JKT189 C.AB547463
48 01B.MY.07.07MYKT021.GQ175883
22 01A1.CM.01.01CM 0001BBY.AY371159
36 cpx.CM.00.00CMNYU830.EF087994
04 cpx.CY.94.94CY032 3.AF049337
37 cpx.CM.00.00CMNYU926.EF116594
11 cpx.CM.95.95CM 1816.AF492624
35 A1D.AF.07.169H.GQ477446
A6.RU.11.11RU6950.JX500694
03 A6B.RU.97.KAL153 2.AF193276
A6.UA.12.DEMA112UA024.KU749403
A6.BY.13.PV85.KT983615
A6.IT.02.60000.EU861977
A1.CD.02.LA01AlPr.KU168256
A3.SN.01.DDI579.AY521629
A1.CM.08.886 24.KP718928
A1.KE.11.DEMA111KE002.KF716474
130 A1B.CY.17.CY397.OP781326
A1.ES.15.100 117.KY496622
A1.RW.11.DEMA111RW002.KF716472
A1.SE.13.078SE.MF373181
A1.PK.15.PK034.KX232624
A1.UG.11.DEMA110UG009.KF716486
CPZ.TZ.06.TAN5.JN091691
0.050
